# Cascade reaction networks within audible sound induced transient domains in a solution

**DOI:** 10.1038/s41467-022-30124-x

**Published:** 2022-05-02

**Authors:** Prabhu Dhasaiyan, Tanwistha Ghosh, Hong-Guen Lee, Yeonsang Lee, Ilha Hwang, Rahul Dev Mukhopadhyay, Kyeng Min Park, Seungwon Shin, In Seok Kang, Kimoon Kim

**Affiliations:** 1 Center for Self-assembly and Complexity (CSC), Institute for Basic Science (IBS), Pohang, 37673 Republic of Korea; 2 Department of Chemistry, Pohang University of Science and Technology (POSTECH), Pohang, 37673 Republic of Korea; 3 Department of Biochemistry, Daegu Catholic University School of Medicine, Daegu, 42472 Republic of Korea; 4 Department of Mechanical and System Design Engineering, Hongik University, Seoul, 04066 Republic of Korea; 5 Department of Chemical Engineering, Pohang University of Science and Technology (POSTECH), Pohang, 37673 Republic of Korea; 6 Present Address: Department of Chemistry, Ramananda College, Bankura University, Bishnupur, 722122 West Bengal India

**Keywords:** Self-assembly, Biocatalysis, Supramolecular chemistry

## Abstract

Spatiotemporal control of chemical cascade reactions within compartmentalized domains is one of the difficult challenges to achieve. To implement such control, scientists have been working on the development of various artificial compartmentalized systems such as liposomes, vesicles, polymersomes, etc. Although a considerable amount of progress has been made in this direction, one still needs to develop alternative strategies for controlling cascade reaction networks within spatiotemporally controlled domains in a solution, which remains a non-trivial issue. Herein, we present the utilization of audible sound induced liquid vibrations for the generation of transient domains in an aqueous medium, which can be used for the control of cascade chemical reactions in a spatiotemporal fashion. This approach gives us access to highly reproducible spatiotemporal chemical gradients and patterns, in situ growth and aggregation of gold nanoparticles at predetermined locations or domains formed in a solution. Our strategy also gives us access to nanoparticle patterned hydrogels and their applications for region specific cell growth.

## Abstract (web summary)

It is difficult to achieve spatiotemporal control over chemical cascade reactions. Here, the authors report on the generation of transient domains in an aqueous medium using sound induced liquid vibrations allowing for control of chemical gradients and patterns and use this to pattern nanoparticles in hydrogels.

## Introduction

The coexistence of different chemical domains in which various chemical cascade reaction networks operate with spatiotemporal control is one of the fundamental principles of operation in living systems, particularly in eukaryotic cells^1–7^. Cells have developed mechanisms for the precise sensing of positional information of biochemical and for regulating the cascade processes in which they are involved. Intracellular gradients of biomolecules arise from the spatial separation of opposing reactions in biochemical reaction cycles. These gradients provide positional cues for executing vital transiently active cellular functions. To understand and mimic such localization and control over biochemical networks in living systems, various artificial counterparts such as liposomes, vesicles, polymersomes, etc. have been developed^8–18^. For instance, amphiphilic block copolymer-based semipermeable polymersomes were synthesized by van Hest and co-workers^12–15^. More recently, coacervate-based biomimetic systems were also extensively studied by Mann and others^19–23^. These systems provide specific domains that allow spatiotemporal control of various cascade reaction networks akin to biological systems. In a different strategy to execute such localization and control over chemical reactions, Pickering emulsions have also been explored^24^. A recent development in this area is the concentric liquid reactors formed under centrifugal force induced out-of-equilibrium conditions and utilized for sequential chemical synthesis and separation by Grzybowski and co-workers^25^. Given the requirements of a prudent molecular design that often leads to cumbersome synthetic steps in the case of the membrane-based systems, and the need for an astute choice of pairwise immiscible liquids in liquid-liquid phase separating membraneless systems, there are large demands for the development of novel straightforward strategies for the creation of programmable domains within a solution where specific chemical reaction networks can be spatiotemporally controlled. Among chemical reactions, enzymatic networks especially have biological significance and possess unique advantages. In general, enzyme cascades can be regulated either by tuning the abundance or activity of the enzymes or by perturbing the enzymatic reaction network with small molecules acting as inhibitors or competitors. However, it remains a central objective of synthetic biologists and chemists to explore alternate strategies to spatiotemporally control such enzymatic reaction networks, to program the predictable formation of chemical gradients through the spatiotemporal control of their reaction kinetics.

An external physical stimulus like light energy, which can be locally applied and can be remotely controlled, has been a useful tool to control the spatiotemporal assembly of organic molecules and that of nanoparticles within a matrix where chemical gradients are generally controlled by diffusion rather than by convection. In a recent work, Prins and co-workers demonstrated that kinetic asymmetry could emerge even at the macroscopic level within a gold nanoparticle embedded hydrogel matrix stimulated by a spatial delivery of light energy thereby installing a non-equilibrium state^26^. Nevertheless, controlling the kinetics of nanoparticle formation and their self-assembly utilizing localized energy delivery in a solution still remains challenging due to the uncontrolled diffusion and convectional currents operating within the system.

Sound energy, which can induce vibrations within a medium while traveling through it and has found wide applications in physics, medical science, materials science and other fields^27–29^. However, the use of sound energy in chemistry is mostly limited to ultrasound-induced cavitation effects that can efficiently execute several chemical reactions^30–32^. Nevertheless, utilizing audible sound (in the range of 20–20,000 Hz) to control any molecular or supramolecular events and synthesis is still in its infancy, most likely due to its low intensity, which is unable to induce chemical transformations^33^. However, it has been long known that vertical shaking of a liquid-filled dish using audible sound (or a vibration generator) can generate a standing wave pattern on the liquid surface (Faraday instability), and the patterns change significantly depending on the frequency of the applied sound and the shape of the dish^34–38^. Based on this phenomenon, very recently we have developed a facile strategy that utilizes audible sound to control the spatiotemporal distribution of chemical components in out-of-equilibrium systems resulting in the formation of predictable chemical patterns^39^. We specifically utilized the audible sound-induced vibration of the air-water interface to promote the dissolution of atmospheric gases (e.g., oxygen and carbon dioxide) to obtain spatiotemporal control over redox or pH-specific chemical reactions, which were easily visualized as predicable and programmable spatiotemporal patterns. The patterns consisted of distinct redox-specific and/or pH-specific domains, which were visualized from the color of the chemical indicators present in the solution. These domains although transiently generated, retained their position and distinct boundary features for a specific time period^40^. Therefore, we anticipated that redox or pH-responsive chemical reactions can favorably occur within these transient domains during the pattern formation process. The spatiotemporal execution of more complex reaction systems such as multistep enzymatic cascade reactions and controlling their kinetics within these transient domains was another interesting challenge that we considered exploring.

**Fig. 1:**
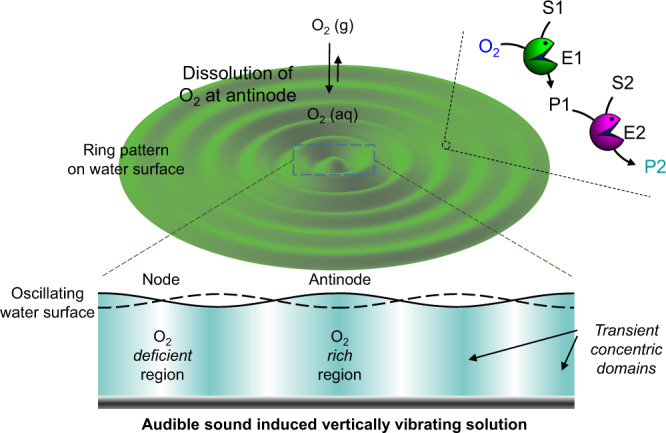
Audible sound induced generation of transient domains and spatiotemporally controlled cascade reaction networks. Schematic of the audible sound induced surface standing wave and generation of corresponding concentric rings (cross-sectional view). Spatiotemporally controlled cascade reaction of substrate S1 with an oxygen responsive enzyme E1 is facilitated at the antinodal domains due to the region-specific dissolution of oxygen. The final product P2 is generated by the reaction of product P1 and substrate S2 with enzyme E2, which is observed as a color pattern.

Herein, we report spatiotemporal control of enzyme-mediated cascade reactions within audible sound-induced transient domains created in a solution without utilizing any structure inducing molecules (Fig. 1). This is further extended to the spatiotemporal control over the in situ growth and self-assembly of nanoparticles within predictable domains present in the solution. Additionally, the preparation of nanoparticle-patterned hydrogels and their utilization for region-specific growth of cells is also explored. Our approach will offer a promising strategy to control chemical processes within predictable yet transiently generated domains within a solution. The strategy can be further utilized to gain control over the region-specific reaction kinetics of enzyme cascade reactions and other complex chemical network systems in solution. The present findings will be significant in the development and control over cascade reaction networks in a solution.

## Results

### Cascade reactions within audible sound induced transient domains

Among enzyme cascade reactions, we chose the widely studied glucose oxidase (GOx) and horseradish peroxidase (HRP) cascade combination with glucose and a redox responsive colorimetric dye. Dyes such as 2,2’-azino-bis(3-ethylbenzothiazoline-6-sulfonic acid) (ABTS) and *o*-dianisidine are typically utilized as substrates in the aforementioned cascade. Recently, Hess and co-workers have reported the spontaneous generation of colored spatiotemporal patterns of ABTS radical utilizing the same enzymatic cascade system that arose from reaction-driven Rayleigh–Bernard convection^41^. Although the overall nature of the resulting patterns was rather similar in each trial, the patterns were not exactly the same in general. Nevertheless, predictable pattern generation depends on the region-specific control of oxygen (substrate) dissolution into the solution, which as mentioned before can be efficiently controlled by audible sound induced liquid vibrations^39^.

As shown in Fig. 1, during the audible sound induced vertical vibration of the liquid, the atmospheric oxygen molecules undergo region-specific dissolution and diffusion into the solution. Specifically, the nodes where the vibration is minimum show lesser gas dissolution (oxygen deficient region) while at the antinodal positions where the vibration is maximum the gas dissolves more efficiently into the solution (oxygen rich region). The nodal and antinodal positions are reproducibly obtained at the same places in the solution for all trials and as a consequence, the colored spatiotemporal patterns of ABTS radical obtained by this strategy should be the same or predictable over several repeated cycles. The overall solution in the dish is in a way isolated into domains differing in their oxygen concentration (oxygen rich: intense bluish cyan and oxygen deficient: pale cyan). These distinctly colored chemical gradients are generated due to the regional expedited dissolution of atmospheric gases such as oxygen into certain concentric domains within the solution, which retain their identity, position and boundary features transiently in solution and can be utilized to execute enzymatic cascade reactions within a single phase with much more precision.

**Fig. 2:**
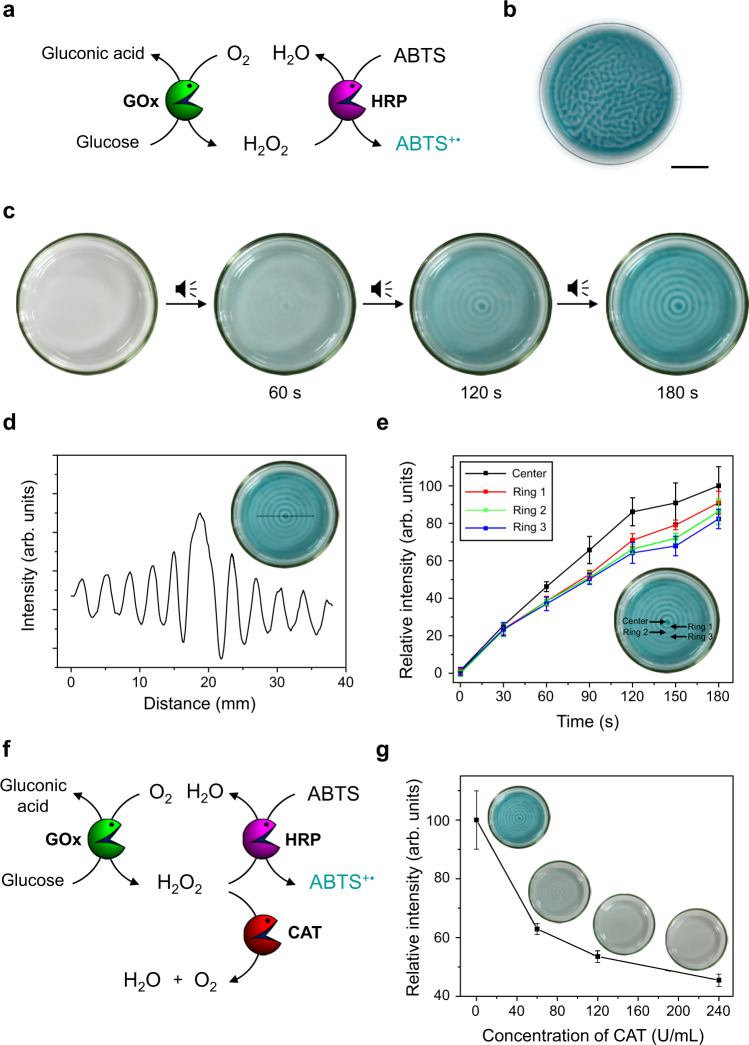
Audible sound mediated spatiotemporal control over glucose/GOx/HRP/ABTS cascade reaction. **a** Schematic representation of glucose/GOx/HRP/ABTS cascade reaction. Here the color pattern is generated from production of the cyan-colored ABTS^+•^. GOx: glucose oxidase, HRP: horseradish peroxidase, ABTS: 2,2’-azino-bis(3-ethylbenzothiazoline-6-sulfonic acid). **b** Random shaped pattern generated without applying audible sound (scale bar is 1.5 cm). **c** Time-dependent changes of a concentric ring pattern obtained by applying an audible sound input (40 Hz). **d** A line profile of image color intensity for the color pattern taken at 3 min. **e** Time-dependent changes in the image color intensity with standard deviation (*N* = 10) at the central region of the pattern and the first three antinodal concentric domains. **f** Schematic of the enzymatic cascade reaction with catalase as a competitive scavenger for H_2_O_2_. CAT: catalase. **g** Effect of catalase for the circular ABTS^+•^ pattern generation. The color intensities with standard deviation (*N* = 10) at the center of the patterns were compared. Inset: pattern images taken after 3 min of reaction.

The experimental setup used in the present study is shown in Supplementary Fig. 1. A schematic representation of the GOx-HRP enzymatic cascade is portrayed in Fig. 2a. The typical reaction involves the ping-pong mechanism of GOx where the first step is the reduction of GOx-FAD to GOx-FADH_2_ by glucose. The recovery of reduced GOx-FADH_2_ was initially taken care of by oxygen and produces H_2_O_2_, which is further used by HRP in order to oxidize ABTS (colorless) to ABTS^+•^ (cyan)^41^. The pattern experiments were performed after optimizing the concentration of the cascade ingredients with a solution (5.0 mL) containing GOx (3.6 U/mL), HRP (2.2 U/mL), ABTS (1.0 mM), and glucose (50 mM) taken in a well-cleaned glass Petri dish, which was tightly covered. The dish was then placed on a loudspeaker connected to a function generator and waited until the solution became colorless. When the solution was exposed to air, in the absence of any external sound input, a randomly shaped cyan-colored pattern was generated as shown in Fig. 2b. The same pattern was not reproducibly obtained in the subsequent trials. In contrast, when the solution was exposed to air in the presence of an audible sound input (40 Hz), a “target” (or concentric ring) pattern was formed, as shown in Fig. 2c. Within a few seconds of audible sound induced vibration of the enzyme-substrate mixture, a concentric ring pattern with cyan and colorless domains was generated due to the region-specific preference in the dissolution of oxygen and subsequent enzymatic cascade reactions. More specifically, the cyan-colored regions correspond to the oxygen rich antinodes where H_2_O_2_ is produced by the O_2_/glucose/GOx reaction, followed by the generation of ABTS^+•^ by the H_2_O_2_/ABTS/HRP reaction. On the other hand, the colorless regions correspond to the nodes where the formation of ABTS^+•^ is limited due to the minimal vibration of liquid and less oxygen uptake. In the present case, since the cyan-colored regions are separated by the colorless regions, the contents of each cyan-colored region are not mixed with those of the adjacent regions. Accordingly, the cyan-colored domains can be considered as “pseudo-compartments”, while the colorless regions can be thought of as “pseudo-barriers” as they spatially separate transient domains in the solution. Although the boundaries of the transient pseudo-compartments generated in this way are less clearly defined than traditional permanent compartments based on lipids or other structure inducing molecules, they could be utilized to generate regional chemical concentration gradients and successfully control chemical reactions.

The strategy also works well in the case of other substrates as evident from a similar pattern with orange and colorless domains that were generated in case we used *o*-dianisidine as a colorimetric indicator (Supplementary Fig. 2). The effect of atmospheric oxygen concentration, which plays a key role in the aforementioned pattern generation process was also tested. For example, no pattern was generated under inert conditions, while the pattern generation was faster in the presence of an oxygen rich atmosphere than under normal atmospheric conditions. (Supplementary Fig. 3).

The formation of the target pattern here is presumably due to the viscous nature of the solution with weaker vibration, which suppresses the lateral flow thereby generating a concentric ring-based pattern rather than a two-vortex pattern as previously observed^39,42^. The cyan-colored concentric domains remain transiently stable in the solution. However, after ~30 min, most of the solution turns into a cyan color, which can be ascribed to the slow diffusion of H_2_O_2_ throughout the solution. Since the standing wave generated in the dish is highly dependent on the applied audible sound frequency, we could control the pitch of the colored ring pattern as shown in Supplementary Fig. 4, thereby we can specifically change the distance between the concentric domains, which can be a useful strategy to affect the chemical diffusion or communication between these domains. In addition, the number of domains increases with an increase in the diameter of the dish as shown in Supplementary Fig. 5. The shape of the transient domain, that is, the cyan-colored features in the pattern, can also be changed by changing the shape of the dish. For example, a target pattern is observed in a circular dish, while a checkerboard pattern was observed in a square dish (Supplementary Fig. 6). To further establish the predictability and reproducibility of patterns developed by our strategy, we performed the pattern formation experiments by varying the depth of the solution with and without applying audible sound (Supplementary Fig. 7). Thereby, we surmised that under the typical experimental conditions, only concentric ring domains were generated in the presence of audible sound, and we could clearly suppress the effects of the reaction-driven Rayleigh–Bernard convection usually observed in these systems under normal circumstances. We observed that during the pattern formation experiments, once a random pattern is generated in the absence of audible sound, it is difficult to reorganize the chemical gradients to obtain a pure concentric pattern even if the sound is applied later, as shown in Supplementary Fig. 8. The resultant pattern is therefore a mixture of random maze-like and concentric ring-shaped chemical gradients.

Another way of interpreting the patterns is through the rate of the enzyme cascade reaction, which is comparatively much faster at the antinodes than at the nodal positions. The rate of reaction even varied at the individual antinodal domains. Image analysis of the patterns at any particular instant during the pattern generation showed that the color intensity corresponding to the amount of ABTS^+•^ formation showed a steady decrease as one moved from the center to the periphery (Fig. 2d). This observation can be attributed to the rate of oxygen dissolution being affected by the concomitant decrease in the amplitude of the vibrations at the antinodal positions from the center towards the periphery, which is well established in the literature^43^. As a matter of fact, we monitored the time-dependent changes in the ABTS^+•^ formation at the center and at the first three concentric ring-shaped domains (at the antinodes), where we clearly observed that the rate of progression of the enzyme cascade reaction is different at the different concentric rings being highest at the center and shows a gradual decline as the rings are placed towards the periphery (Fig. 2e). The overall rate of pattern formation or the rate of the enzyme cascade reaction in the specified domains can also be manipulated by the addition of a competitor. For instance, the addition of catalase leads to the fast decomposition of H_2_O_2_ and thereby affects the rate of ABTS^+•^ formation (Fig. 2f)^44^. We observed that the rate of enzyme cascade reaction at the concentric rings was significantly decreased and the pattern formation was largely delayed as we increased the catalase concentration in solution (Fig. 2g).

### In situ growth of gold nanoparticles within audible sound induced transient domains

**Fig. 3:**
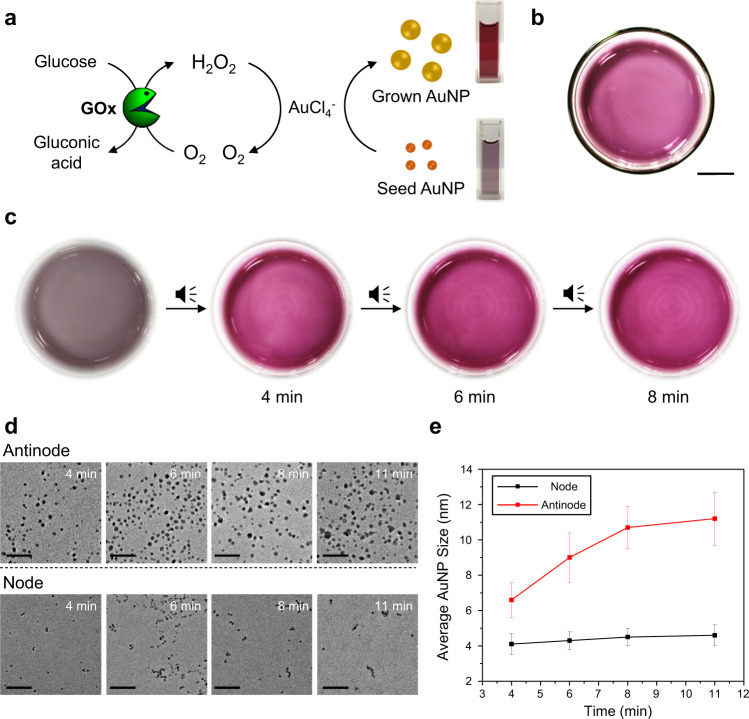
Audible sound mediated spatiotemporal control over glucose/GOx/AuCl_4_^-^/seed AuNP cascade reaction. **a** Schematic representation of glucose/GOx/AuCl_4_^-^/seed AuNP cascade reaction. Here the colored pattern is generated from the in suit grown AuNPs. GOx: glucose oxidase, AuNP: gold nanoparticle. **b** Pattern generated without applying audible sound (scale bar is 1.5 cm). **c** Time-dependent changes of circular pattern generation with applying 30 Hz of audible sound. **d** TEM images of AuNP solutions taken at the antinodal (top) and nodal (bottom) positions of the colored pattern at 4, 6, 8, and 11 min, respectively (scale bar is 50 nm). **e** Plots for the time-dependent changes in the average size with standard deviation (*N* = 100) of the nanoparticles obtained from the TEM images from samples at the antinodal and nodal positions of the solution, respectively.

After the successful control of the GOx-HRP enzymatic cascade, we decided to expand our strategy to spatiotemporally control the in situ seeded growth of gold nanoparticles (AuNPs) (Fig. 3a) within the audible sound generated transient domains within the same solution. Here, we utilized the growth of small sized AuNPs (~4 nm, acting as seed) in a cascade with the biocatalytic reaction between glucose, GOx and O_2_ as schematically represented in Fig. 3a^45–47^. Seed AuNPs were prepared by literature procedure and characterized by transmission electron microscopy (TEM) and UV–Vis spectroscopy. The UV–Vis spectra of the as synthesized seed AuNPs showed characteristic surface plasmon peak at 513 nm, which supported the formation of small sized seed and the TEM experiments further confirmed the formation of spherical seed AuNPs with an average size of 4.1 nm (Supplementary Fig. 10). For the in situ growth of AuNPs, we varied different parameters and optimized a suitable condition for executing the audible sound experiments (Supplementary Fig. 11). When an aqueous solution (5.0 mL) containing seed AuNP (8 nM), GOx (35 U/mL), glucose (50 mM), and AuCl_4_^-^ (0.6 mM) was placed in a Petri dish, the solution turned wine red in color within 10 min (Fig. 3b), indicating the formation of larger AuNPs (average size ~ 11 nm) due to the reduction of Au^3+^ to Au^0^ on the seed AuNP surface by the reaction byproduct H_2_O_2_. The results obtained from time-dependent UV–Vis spectra corroborates the AuNP growth process in the solution state (Supplementary Fig. 12).

When the same cascade biocatalytic nanoparticle growth mixture was subjected to audible sound and exposed to the air, we observed the appearance of red-colored concentric ring pattern within a few minutes, indicating the spatiotemporal expedited growth of AuNPs at the antinodal regions, as shown in Fig. 3c. We observed that the more oxygen supplied antinode regions turned wine red within a few minutes owing to the accelerated production of H_2_O_2_ that promoted the growth of the seed AuNPs, while the nodal positions where the supply of oxygen was limited appeared as thin concentric lines with a comparatively less intense wine red color. From the time-dependent TEM images obtained from aliquots extracted from each area, we further confirmed the facilitated growth of the AuNPs at the transiently generated domains placed at the antinodal positions of the standing wave. Whereas, a time-dependent TEM analysis of samples extracted from the nodal positions showed minimal changes in the size of AuNPs (Fig. 3d, e). Although our audible sound method allowed us to control the in situ regional growth of AuNPs within transiently generated domains in a solution, a sharp boundary between the concentric ring-shaped domains (at the antinodal positions) as in the case of ABTS/ABTS^+•^ pattern was not achieved in this case, which can be ascribed to relatively small differences in molar extinction coefficients (~100-fold) as well as surface plasmon peaks (~20 nm) between seed and fully grown AuNPs^48^. Therefore, we aimed towards exploring a nanoparticle-based cascade reaction with a clear contrast between the concentric ring-shaped domains. We anticipated that the large difference in solution color between dispersed and aggregated AuNPs may be useful in obtaining such results. It may also simultaneously provide an easy route for the spatiotemporal self-assembly of specifically surface functionalized AuNPs.

### Gold nanoparticle aggregation within audible sound induced transient domains

**Fig. 4:**
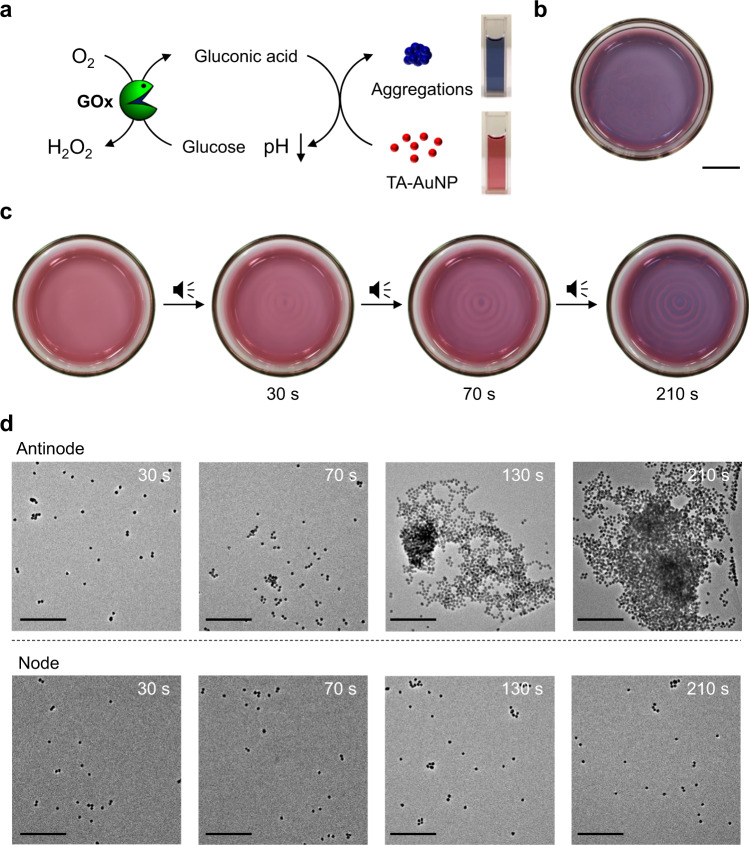
Audible sound mediated spatiotemporal control over glucose/GOx/TA-AuNP cascade reaction. **a** Schematic representation of glucose/GOx/TA-AuNP cascade reaction. Here the color pattern is generated from the blue-colored aggregates of TA-AuNPs. GOx: glucose oxidase, TA-AuNP: thioctic acid-functionalized gold nanoparticle. **b** Random shaped pattern generated without applying audible sound (scale bar is 1.5 cm). **c** Time-dependent changes of a concentric ring pattern generated with applying 30 Hz of audible sound. **d** Time-dependent TEM images of TA-AuNP solution taken at the antinodal (top) and nodal (bottom) positions of the colored pattern at 30, 70, 130, and 210 s, respectively (scale bar is 200 nm).

On demand control of metal nanoparticle assembly is important as the physical properties of metal nanoparticles are largely dependent on the aggregation or stacking of individual nanoparticles. Controlling such nanoparticle assembly in a spatiotemporal fashion is challenging and has so far been achieved using external stimuli such as chemicals, biomolecules, light, magnetic field, etc^49–52^. To apply our audible sound method to spatiotemporally program a nanoparticle assembly, we prepared carboxylic acid-functionalized gold nanoparticles (AuNPs) because their aggregation tendency and the resulting vivid color changes in acidic pH have been well-known for decades. We first synthesized 13 nm AuNPs capped with citric acid and the surface capping agent of these AuNPs was further exchanged with thioctic acid by ligand exchange reaction as the latter provides better stability and pH response in water^53,54^. As shown in Supplementary Fig. 13, the thioctic acid-functionalized AuNPs (TA-AuNP) were well dispersed in basic and neutral aqueous solution and exhibited a red-wine color. However, it turned blue below pH 4, indicating AuNP aggregation. Therefore, based on the color changes, we can easily recognize the formation of TA-AuNP aggregates under acidic conditions (Fig. 4a).

Following the previous ABTS pattern experiment, here we used the glucose/GOx reaction again because the reaction produces gluconic acid that lowers the pH of the solution. Firstly, TA-AuNP was mixed with glucose/GOx and the solution was exposed to air without applying sound. As the enzymatic reaction proceeds, the pH of the solution decreases and thus TA-AuNP aggregation occurs, resulting in randomly positioned blue regions in the red-colored solution as shown in Fig. 4b. The time-dependent UV–Vis spectra of this solution are given in Supplementary Fig. 14, which establishes the gradual self-assembly of the AuNPs. In contrast, the application of audible sound to the above solution produced a concentric ring pattern with blue and red concentric rings as shown in Fig. 4c. Such a ring pattern was evolved because of the enzymatic reaction, which proceeded preferentially at the antinodal regions transiently generated in the solution. In this pattern, small portions of the red and blue regions were extracted and analyzed by time-dependent TEM analysis. As shown in Fig. 4d, the TA-AuNPs in the red-colored nodal positions of the standing wave, mostly existed as individual particles, while the nanoparticles in the blue-colored concentric regions located at the antinodal positions exhibited the formation of larger nanoparticle aggregates and clusters with time as we expected. Since the color of the dispersed and aggregated nanoparticles was distinctly different, we could track the relative rates of TA-AuNP aggregation at the various antinodal regions through analysis of pattern images exhibited in Fig. 4c. The obtained results clearly corroborated that the TA-AuNP aggregation follows a much faster kinetics at the center and the rate of aggregation gradually decreases as one shifts to the antinodal regions (ring 1 to ring 3) towards the periphery (Supplementary Fig. 15) as observed in the case of the ABTS^+•^ pattern (Fig. 1e). These results confirm that our audible sound method provides an innovative strategy to program the self-assembly of gold nanoparticles within transient domains generated in a solution.

### Nanoparticle-patterned hydrogels for region-specific cell growth

**Fig. 5:**
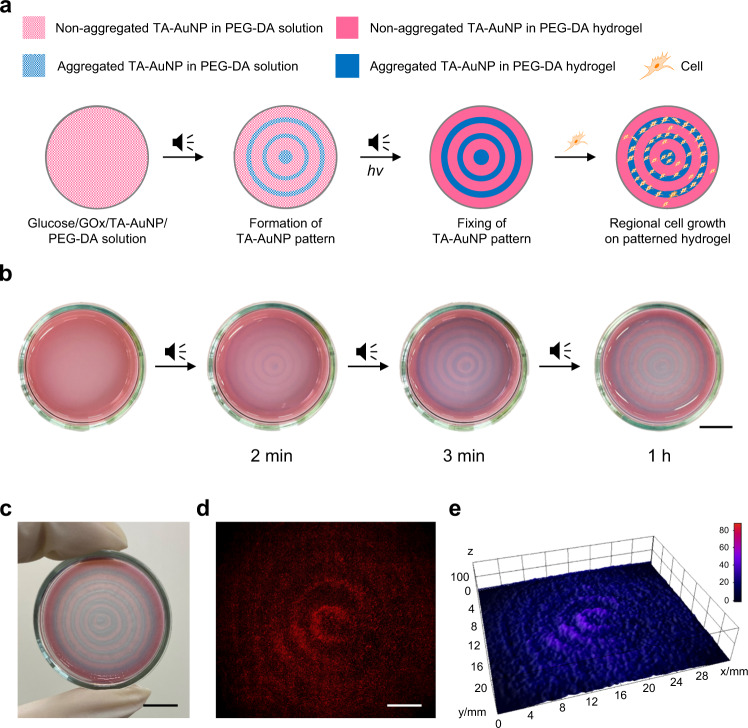
Preparation of TA-AuNP-patterned hydrogel and its application to region-specific cell growth. **a** Schematic representation of sound assisted patterned hydrogel formation and selective cell growth. GOx: glucose oxidase, TA-AuNP: thioctic acid-functionalized gold nanoparticle, PEG-DA: poly(ethylene glycol)-diacrylate. **b** Time-dependent images during the formation of a concentric ring pattern obtained by applying 40 Hz of audible sound and preparation of patterned hydrogel (scale bar is 10 mm). **c** A photograph of the patterned hydrogel (scale bar is 10 mm). **d** HeLa cell growth pattern on the hydrogel (scale bar is 5 mm). **e** 3D fluorescence intensity plot of the region-specific cell growth pattern.

Region-specific growth of cells on specifically designed or patterned substrates can be exploited in a variety of biomedical applications, including regenerative medicine, tissue engineering, development of biosensors, and elucidation of cell-matrix interactions, etc^55,56^. To explore such applications, patterned surfaces of functional polymer or hydrogels have been often utilized as platforms for cell growth^57^. Similarly, the scope of studying the interaction of cells with patterned substrates containing materials such as carbon nanotubes^58^, metal nanoparticles^59^, etc. remains open for investigation. For instance, inhibition of cell growth has been reported in the presence of dispersed AuNPs. Whereas, an increase in the growth rate of cells has been observed in the presence of large AuNP aggregates^59^. The patterns obtained in the aforementioned cases mostly utilize chemical vapor deposition or techniques such as inkjet printing. We thought that using our audible sound-based strategy it may be possible to obtain patterns of AuNPs and AuNP aggregates, which can be further immobilized within a hydrogel matrix. We anticipated that these patterned substrates can be utilized as a platform to obtain region-selective cell growth, which in a way can extend the cascade chemical processes to the next higher level of biological applications. Figure 5a shows a schematic illustration of the nanoparticle-patterned hydrogel preparation and its application as a platform for selective cell growth. When 40 Hz of audible sound was applied to a solution containing TA-AuNP (8 nM), GOx (80 U/mL), glucose (50 mM), poly(ethylene glycol)-diacrylate (PEG-DA) (*M*_n_ = 700, 10 wt%) and a photo-initiator (Irgacure 2959, 1 wt%), spatiotemporal patterns consisting of blue concentric rings of aggregated AuNPs were developed within 3 min as shown in Fig. 5b. The fully developed pattern was fixed by irradiating the solution with 365 nm UV light. Figure 5c shows a photograph of the resulting nanoparticle-patterned hydrogel material. The hydrogel comprising of aggregated (blue region) and non-aggregated (pink region) nanoparticles containing domains was further utilized as a scaffold for region-selective growth of HeLa cells. To improve cell adhesion and proliferation, the hydrogel surface was treated with a cyclic RGDyK peptide and poly-l-lysine conjugate (c(RGDyK)-PLL) (see Methods section)^60,61^, and the resulting hydrogel was incubated overnight after being treated with HeLa cells. When the patterned hydrogel was observed under a fluorescent microscope after 24 h, patterned cell growth is observed as shown in Fig. 5d, and the corresponding three-dimensional (3D) fluorescence image intensity plot of the cells (Fig. 5e) was found to be prominent along with alternate concentric rings as observed in the hydrogel pattern (See also Supplementary Fig. 16). Additionally, cells in the antinodal region show well spread morphology, whereas those in the nodal region do not (Supplementary Fig. 17). Analyzing these images, we could confirm a region-specific cell growth over the AuNP aggregated domains, which coincide with the antinodal positions of the pattern. As a control experiment, we carried out the same study using a hydrogel, random-patterned with AuNPs and AuNP aggregates that was prepared without applying sound. This resulted in random cell growth over the hydrogel surface, as shown in Supplementary Fig. 18. We further confirmed that our strategy of obtaining region-selective cell growth can be extended to other cells, such as human umbilical vein endothelial cells (HUVECs) (Supplementary Fig. 19).

## Discussion

We have demonstrated the utilization of audible sound induced liquid vibration to generate transient domains within a solution wherein enzyme-mediated cascade chemical reactions can be spatiotemporally controlled to obtain predictable and reproducible spatiotemporal chemical gradients and colored patterns. Our approach further provided a way to gain spatiotemporal control over the in situ growth or aggregation of AuNPs in an aqueous solution. Finally, we prepared nanoparticle-patterned hydrogels and showed their utilization for region-specific cell growth. The uniqueness and expandability of our strategy of using audible sound to generate transient domains, which act as pseudo-compartments within a solution, may provide more insights for gaining better control over complex chemical reaction networks at the macroscopic level and in the development of smart materials or systems that can be controlled by such cascade chemical reaction networks.

## Methods

### Materials and general methods

All the reagents and solvents employed were commercially available and used as supplied without further purification. Cyclic RGDyk peptide was obtained from PEPTRON (Daejeon, Republic of Korea). HeLa and HUVEC were purchased from ATCC. Deionized water with a resistivity of 18.2 MΩ cm^−1^ was used to prepare aqueous solutions. UV–visible absorption spectra were collected on a Cary series UV–Vis–NIR spectrophotometer, Agilent Technologies. TEM images were recorded on an FEI Titan Themis electron microscope operating at 300 kV. A function generator (AFG-2005, GW Instek) and a speaker (PC83-8 or DS135-8, Dayton Audio) were used to generate and control vertical vibrations. Vibrational acceleration was measured by a vibration meter (ST-140, Tenmars). Photos of the experiments were taken by a smartphone or a digital camera. A fluorescence microscope (Eclipse Ti-E, Nikon) was used for the imaging of cells.

### Protocol for sound induced transient domains and colored pattern generation experiments

A circular glass Petri dish was mounted on top of a loudspeaker with a flat acrylic tray and the loudspeaker was connected to a function generator to generate vertical sinusoidal vibration. The typical experimental set up is shown in Supplementary Fig. 1. The ranges of frequency and amplitude of vibration were controlled by a function generator and the amplitude of vibration was measured with a vibration meter. For the audible sound-induced transient domains and color pattern generation experiments, frequency in the range of 30−90 Hz with an amplitude of the vibration in the range of 0.20−0.25 g were found to be suitable. Unless otherwise noted, a 56 mm-sized (inner diameter) circular glass Petri dish was used for pattern generation experiments. Each pattern generation experiment was repeated more than 10 times to confirm the reproducibility of the pattern formation process.

### Glucose/GOx/HRP/ABTS cascade reaction without applying sound

A 5.0 mL solution containing 3.6 U/mL GOx, 2.2 U/mL HRP, 1.0 mM ABTS, and 50 mM glucose in PBS (pH 7.1) was purged with nitrogen gas for 1 h and then gently poured into a Petri dish placed on a tray, and covered with a piece of plastic wrap. After the solution turned colorless, the Petri dish was gently shaken to ensure that the solution was homogenous. After waiting for several seconds for further stabilization, the Petri dish was uncovered and exposed to the air to initiate pattern generation. The pattern generation in the dish was recorded with a smartphone.

### Glucose/GOx/HRP/ABTS cascade reaction with applying sound

A 5.0 mL solution containing 3.6 U/mL GOx, 2.2 U/mL HRP, 1.0 mM ABTS, and 50 mM glucose in PBS (pH 7.1) was purged with nitrogen gas for 1 h, and then gently poured into a Petri dish placed on an acrylic tray over the loudspeaker, and covered with a piece of plastic wrap. After the solution turned colorless, the Petri dish was gently shaken to ensure that the solution was homogenous. After waiting several seconds for further stabilization, the Petri dish was uncovered and exposed to the air and the function generator connected to the loudspeaker was turned on to initiate pattern generation. The pattern generation in the dish was recorded with a smartphone. In experiments to study the effect of catalase on color pattern generation, catalase was additionally added to the initial reaction mixture at different concentrations. Pattern experiments were carried out at different concentrations of atmospheric oxygen; a 5 L glass beaker (filled with pure oxygen or argon gas) was used as a reaction chamber for such a purpose (Supplementary Fig. 3). For the pattern generation experiments with different sized or shaped dishes, the solution volume was adjusted in such a way that the height of the solution remain 2 mm, taking into account the size of the dish (Supplementary Figs. 5 and 6).

### Glucose/GOx/HRP/dianisidine cascade reaction with applying sound

A 5.0 mL solution containing 7.2 U/mL GOx, 4.5 U/mL HRP, 2.0 mM *o*-dianisidine, and 50 mM glucose in PBS (pH 7.1) was purged with nitrogen gas for 1 h, gently poured into the Petri dish on a tray. After waiting several seconds for stabilization, the function generator was turned on to initiate the color pattern generation. The reaction scheme and colored pattern in the dish were shown in Supplementary Fig. 2.

### Colored pattern analyses

The images obtained from the pattern experiments were analyzed using ImageJ software. Firstly, the RGB color pattern images were converted into grayscale representation, then the gray values were collected using the multi-point/point tool. For each image at a particular time, 10 data points were collected from the region of interest. Finally, the mean and standard deviations were calculated and plotted. Since the gray value at *t* = 0 s is considered as a background, the initial gray value was subtracted and a percentage change in gray value was calculated. The error bars in the line profile figures represent the standard deviation of the pattern image intensity.

### Effect of applying frequency

To ascertain the effect of frequency on the transient domain formation, experiments were performed by varying the frequency of the applied audible sound. Throughout the frequency range investigated (30−90 Hz), the shape of the domain was found to be similar but the thickness of the concentric ring-shaped domain became narrow with an increase in the frequency of the applied sound as shown in Supplementary Fig. 4.

### Preparation of seed AuNPs

Seed gold nanoparticles (AuNPs) were prepared according to the literature^62^. In a typical experiment, a 20 mL aqueous solution containing 0.25 mM HAuCl_4_ and 0.25 mM trisodium citrate was prepared in a glass sample vial. Next, freshly prepared ice-cold NaBH_4_ (0.1 M, 0.6 mL) was added under vigorous stirring. Stirring was continued for 30 s and the solution was kept undisturbed for 4 h. The seed AuNP was characterized by UV–Vis spectroscopy and TEM (Supplementary Fig. 10).

### Optimizing the conditions for the growth of seed AuNPs

In order to optimize the concentration of various components in the growth of seed AuNPs, UV–Vis experiments were performed with different concentrations of each component. When we varied the concentration of GOx from 35–85 U/mL (keeping the concentration of glucose = 50 mM, AuCl_4_^-^ = 0.60 mM, seed AuNP = 8 nM), there was a blue shift in the surface plasmon peak (530−538 nm), also the generation of red color was faster in case of using a high concentration of GOx (Supplementary Fig. 11a). This indicated that the growth of seed AuNPs could be tuned by changing the GOx concentration. Upon changing the concentration of glucose from 10 to 50 mM (keeping the concentration of GOx = 85 U/mL, AuCl_4_^–^ = 0.60 mM, seed AuNP = 8 nM), the resulting spectra remained the same, suggesting that the changes in glucose concentration in this region had no significant effect on the growth of seed AuNPs (Supplementary Fig. 11b). Then, the concentration of AuCl_4_^–^ was varied 0.2 − 1.0 mM and keeping concentration of glucose = 50 mM, GOx = 50 U/mL, and seed AuNP = 8 nM. As shown in the Supplementary Fig. 11c, the surface plasmon peak value for higher concentration (1 mM) showed a red shift, revealing the formation large sized nanoclusters (surface plasmon peak at 550 nm). Finally, the concentration of seed AuNPs was also varied (2−8 nM) keeping the concentration of glucose = 50 mM, GOx = 50 U/mL, and AuCl_4_^–^ = 0.60 mM, respectively (Supplementary Fig. 11d). Since the high concentration of seed AuNP produced a red color within a few minutes (surface plasmon peak at 533 nm), it was confirmed that it is suitable for sound-induced pattern experiments. At a low concentration of seed AuNPs, the spectra were broad and the surface plasmon peaks appeared at higher wavelengths as shown in Supplementary Fig. 11d. The corresponding average particle sizes were calculated from the TEM images (Supplementary Figs. 11e–h). By combining all the results, we conclude that 50 mM of glucose, 35 U/mL of GOx, 8 nM of seed AuNPs, and 0.60 mM of AuCl_4_^–^ was an appropriate condition for the spatiotemporal in situ growth of AuNPs using our protocol. Time-dependent UV–Vis spectral changes corresponding to the seeded growth of AuNP are presented in Supplementary Fig. 12.

### Sound-controlled in situ growth of AuNPs

Before the experiment, the cascade components (glucose/GOx/seed AuNPs/AuCl_4_^-^) were degassed individually with nitrogen gas for 1 h. Then, 5.0 mL solution containing 50 mM of glucose, 35 U/mL of GOx, 8 nM of seed AuNPs, and 0.6 mM of AuCl_4_^-^ were quickly transferred into a Petri dish on top of the loudspeaker. After waiting several seconds for stabilization, the function generator connected to the loudspeaker was turned on. Within a few minutes, the mauve colored solution was segregated into two domains due to sound-induced liquid vibrations, and the red-colored pattern generated in the dish was recorded with a smartphone.

### Synthesis and characterization of TA-AuNP

After synthesizing 13 nm-sized gold nanoparticles according to the literature, thioctic acid-functionalized gold nanoparticles (TA-AuNPs) were prepared by ligand exchange method^49,50^. For the ligand exchange of the citrate stabilized AuNPs, an ethanolic solution of thioctic acid (10 mM, 4.0 mL) was added to the citrate stabilized AuNPs solution (40 mL) whose basicity was preadjusted to pH 11, the mixture was then stirred for 18 h in the dark. The solution was then centrifuged for 20 min (at 18,000 × *g*, 10 °C), followed by decantation of supernatants. The precipitated TA-AuNPs were redispersed in water and centrifuged again two times more under the same conditions. The resulting TA-AuNPs were characterized by TEM (Supplementary Fig. 13a), and pH-dependent UV–Vis spectral changes were also observed (Supplementary Fig. 13b). Time-dependent UV–Vis spectral changes with glucose/GOx reaction system are presented in Supplementary Fig. 14.

### Sound-controlled TA-AuNP pattern experiments

Prior to the experiments, all the required ingredient solutions were thoroughly degassed with N_2_ gas for 1 h. Then, 5.0 mL solution containing 40 U/mL GOx, 8 nM TA-AuNP, 50 mM NaCl, and 50 mM glucose was taken into a Petri dish. Once the solution became steady, the function generator connected to the loudspeaker was turned on. The pattern generation in the dish was recorded with a smartphone.

### Preparation of gold nanoparticle-patterned hydrogels

Prior to the experiments, all the required ingredient solutions were thoroughly degassed with N_2_ gas for 1 h. Then, 1.5 mL solution containing 80 U/mL GOx, 8 nM TA-AuNP, 75 mM NaCl, 50 mM glucose, 10 wt% PEG-DA (*M*_n_ = 700), and 1 wt% Irgacure 2959 was taken into a 36 mm-sized Petri dish. Once the solution became steady, the function generator connected to the loudspeaker was turned on and a frequency of 40 Hz was applied to this solution. The pattern generation in the dish was recorded with a smartphone. The fully developed pattern was fixed by irradiating the solution in the Petri dish with a handheld UV lamp (365 nm) for 1 h.

### Preparation of c(RGDyK)-PLL

A cyclic RGDyK peptide and poly-l-lysine conjugate (c(RGDyK)-PLL) was prepared by following the previous work^60,61^. To a solution of poly-l-lysine (PLL) (50 mg) in deionized (DI) water (5 mL), a solution of dibenzocyclooctyne-*N*-hydroxysuccinimidyl ester (100 μL, 0.2 mM in DMSO) was added and the mixture was stirred at room temperature for 1 h. Followed by the addition of cyclo[Arg-Gly-Asp-D-Phe-Lys(Azide)] (c(RGDyK-azide), 100 μL, 0.2 mM in DI water) to the reaction mixture and stirred at room temperature for 12 h. After the reaction mixture was purified by dialysis against water using a membrane with a molecular weight cut-off of 10000, the sample was lyophilized to give c(RGDyK)-PLL (45 mg). The product was dissolved in DI water (1 mg/mL) and used in the following experiments.

### Cell culture experiments with a patterned hydrogel

The patterned hydrogel was thoroughly washed with PBS buffer to remove any unreacted residues. Subsequently, the hydrogel was coated with the solution of c(RGDyK)-PLL for 1 h. A solution of live cells (4 mL, 2 × 10^6^ cells, HeLa in DMEM), which was stained with DiIC18(3), was transferred to the hydrogel. In the case of HUVECs, a solution of live cells (4 mL, 2 × 10^6^ cells, HUVEC in F-12K), which was stained with SP-DiOC18(3). The cells were incubated at 37 °C in a humidified incubator under standard culture conditions for 1 to 3 days. The cells were imaged under a fluorescence microscope and the images were manually arranged to make an overview image of the hydrogel.

### Reporting summary

Further information on research design is available in the Nature Research Reporting Summary linked to this article.

## Supplementary information

Supplementary Information

Reporting Summary

## Data Availability

The authors declare that all the data supporting the findings of this study are available within the paper and its Supplementary Information file or from the corresponding authors upon request.
