# Supplementary material for: Cascade reaction networks within audible sound induced transient domains in a solution
Source: Nat Commun. 2022 May 2;13:2372. doi: 10.1038/s41467-022-30124-x (PMC9061750; doi:10.1038/s41467-022-30124-x)
Supplement: Supplementary file 1 — Supplementary Information [file 41467_2022_30124_MOESM1_ESM.pdf]

Supplementary Information

Cascade reaction networks within audible sound induced transient domains in a solution

Prabhu Dhasaiyan, Tanwistha Ghosh, Hong-Guen Lee, Yeonsang Lee, Ilha Hwang*, Rahul Dev Mukhopadhyay*, Kyeng Min Park, Seungwon Shin, In Seok Kang & Kimoon Kim*

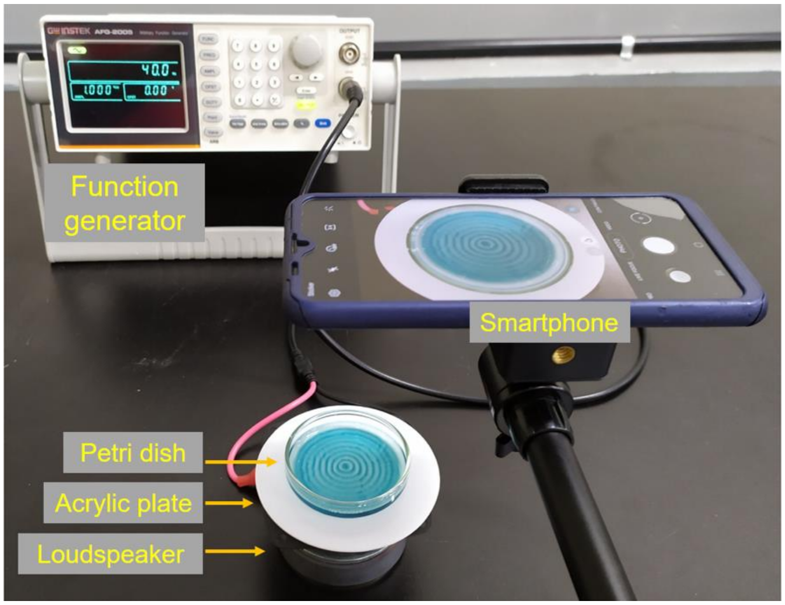

Supplementary Figure 1. A photograph of the experimental setup.

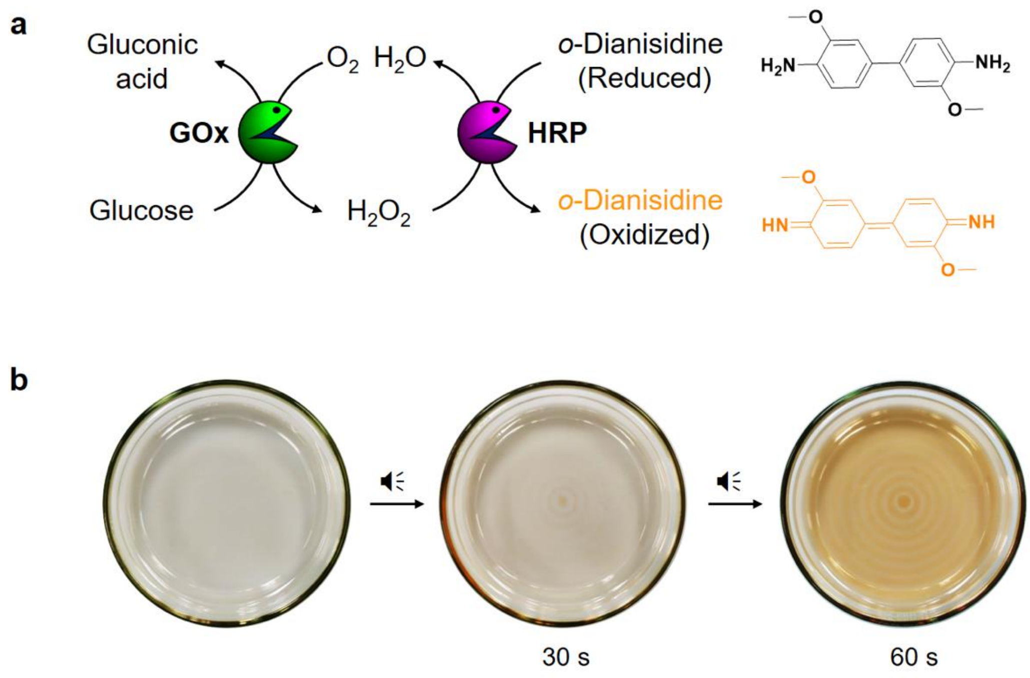

Supplementary Figure 2. Glucose/GOx/HRP/dianisidine cascade reaction with applying sound. a Schematic representation of glucose/GOx/HRP/dianisidine cascade reaction. GOx: glucose oxidase, HRP: horseradish peroxidase. Here the colored pattern is generated from the orange-colored oxidized *o*-dianisidine. **b** Time-dependent changes in circular pattern generation by applying 40 Hz of audible sound.

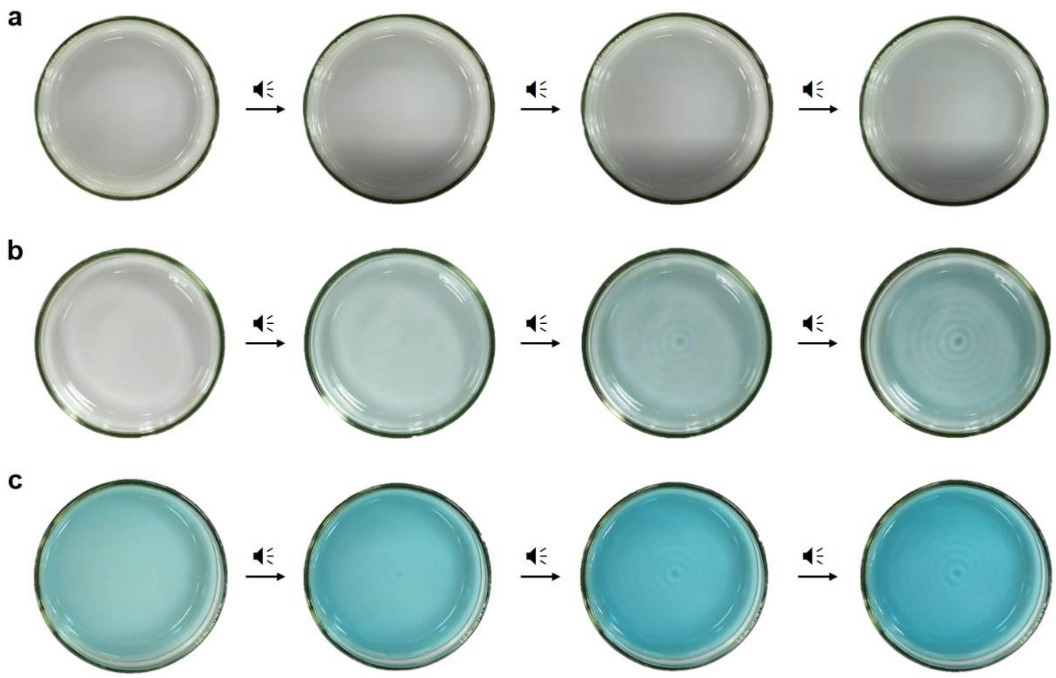

Supplementary Figure 3. Effect of oxygen concentration on pattern generation. Photographs show changes in the ABTS patterns over time (10, 40, 70, and 100 s, respectively) when 40 Hz audible sound is applied under different atmospheric oxygen concentrations. The pattern formation is recorded under **a** inert atmosphere (argon gas filled inside a glass beaker), **b** normal atmosphere (a glass beaker removed), and **c** saturated oxygen atmosphere (inside a glass beaker), respectively.

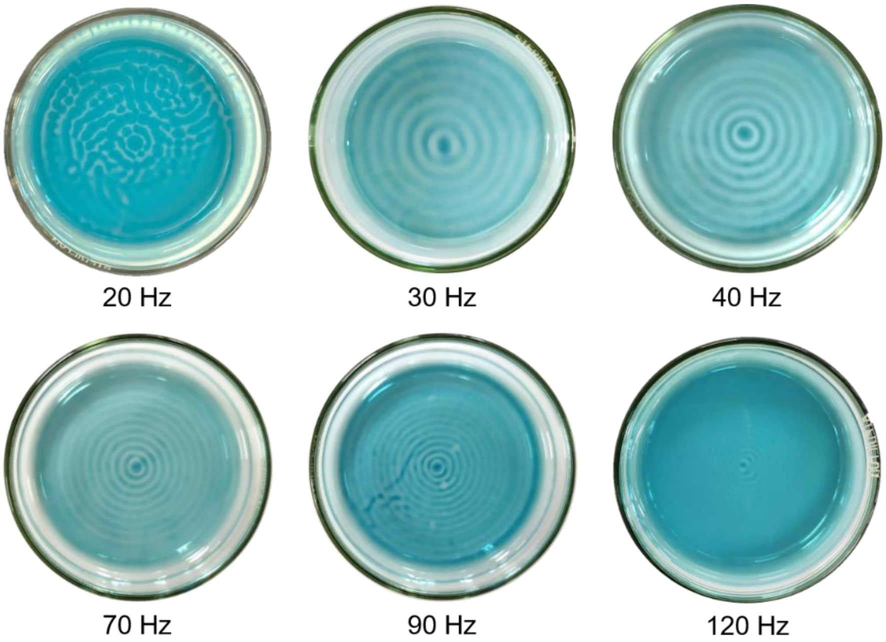

Supplementary Figure 4. Cascade reactions within audible sound frequency-controlled transient domains in a solution. Pattern images of glucose/GOx/HRP/ABTS cascade reaction system obtained at different input frequencies of audible sound. Considering the shape and reproducibility, 30−90 Hz is a suitable range for the formation of the concentric ring patterns under our experimental conditions.

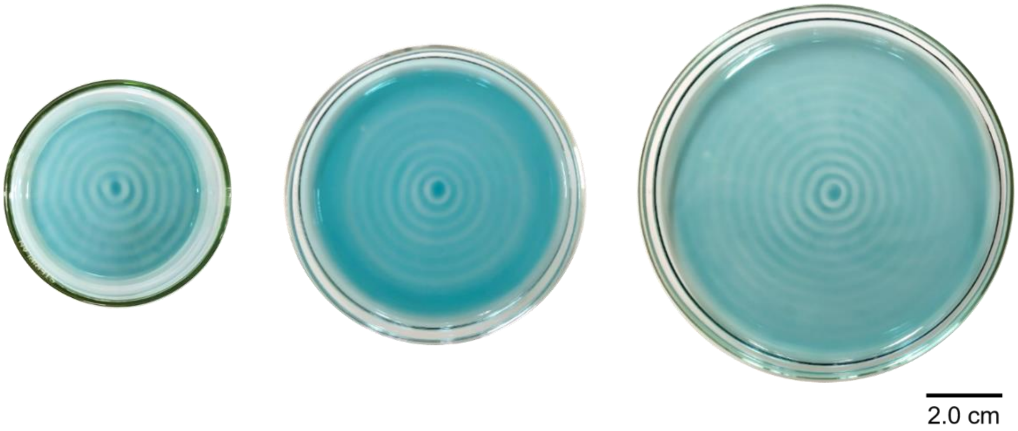

Supplementary Figure 5. Cascade reactions within transient domains in a solution obtained with different sized Petri dishes. Pattern images of glucose/GOx/HRP/ABTS cascade reaction system obtained with different sized Petri dishes by applying 30 Hz of audible sound. The inner diameter of the dishes is 56, 76, and 96 mm in size, respectively.

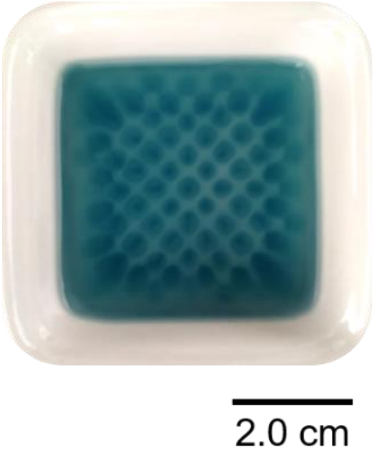

Supplementary Figure 6. Effect of dish shape on pattern generation. A photograph of a checkerboard pattern generated from glucose/GOx/HRP/ABTS cascade reaction system in a square shaped porcelain dish by applying 40 Hz of audible sound.

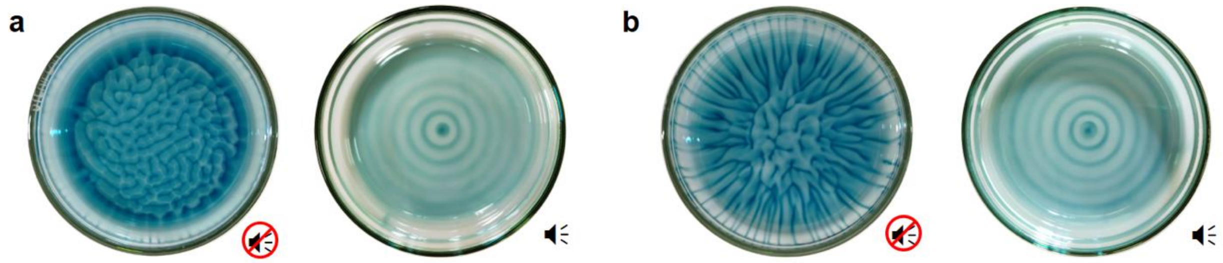

Supplementary Figure 7. The effect of solution height on ABTS pattern. a Pattern images generated with reaction mixture having a depth of 3.5 mm, in the absence (left) and presence (right) of applying 30 Hz audible sound. **b** Pattern images generated with reaction mixture having a depth of 5.0 mm, in the absence (left) and presence (right) of applying 30 Hz audible sound.

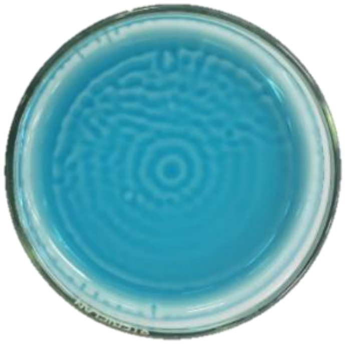

Supplementary Figure 8. Effect of audible sound on the random pattern. A photograph of the pattern was generated when 40 Hz audible sound was applied to a random ABTS pattern (obtained in the absence of sound). The characteristics of the maze-like and concentric ring-shaped domains appear mixed in the resultant pattern.

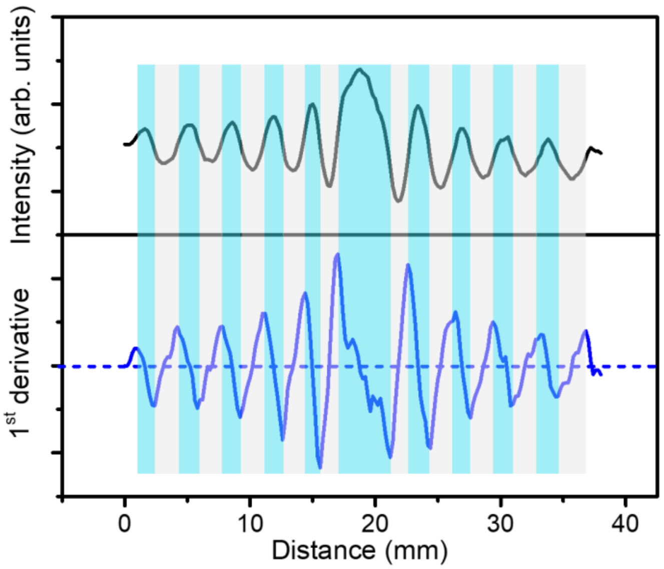

Supplementary Figure 9. Intensity plot analysis of the ABTS pattern. The ABTS pattern intensity plot at 180 s (black line, from Fig. 2d) and its first derivative (blue line). 'Pseudo-compartments' and 'pseudo-barriers' can be defined as the regions between the maximum and minimum points of the first derivative plot, respectively. The pseudo-compartments (cyan regions) and the pseudo-barriers (pale gray regions) appear alternately.

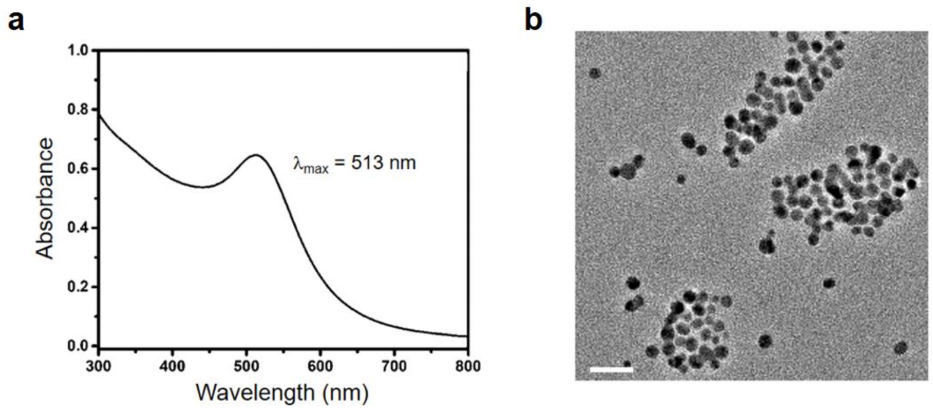

Supplementary Figure 10. Characterization of seed AuNP. a UV-Vis spectrum of seed AuNP. **b** TEM image of seed AuNP (scale bar is 20 nm).

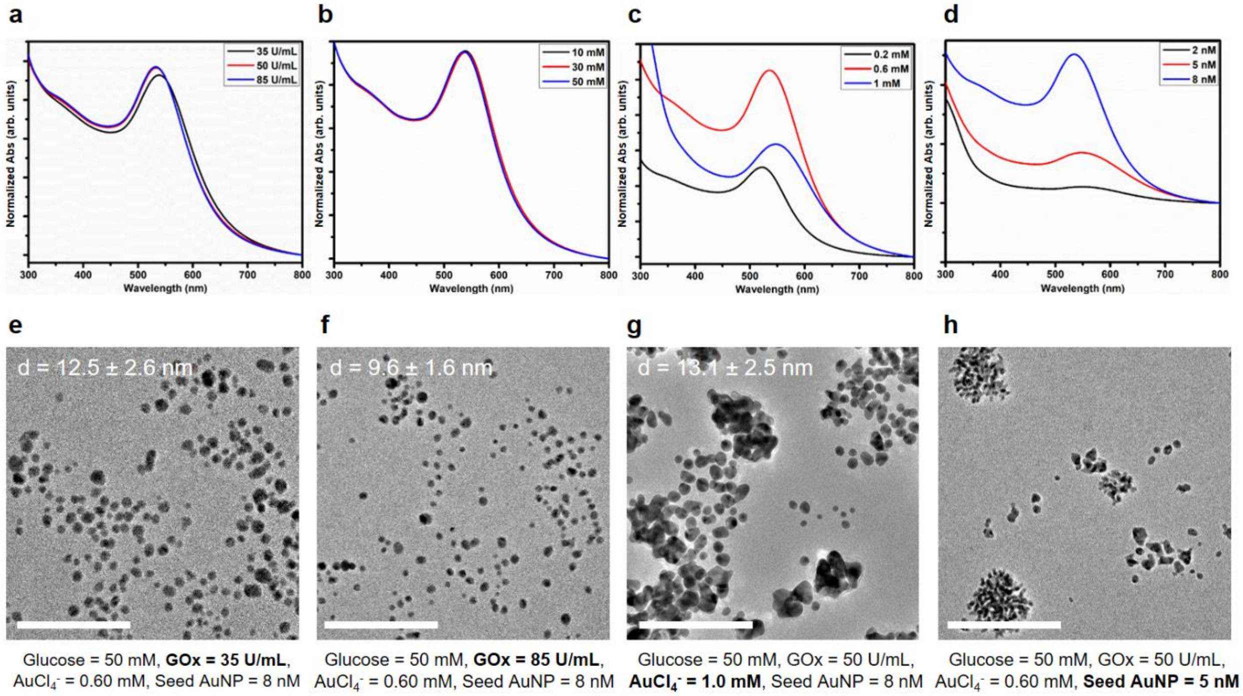

Supplementary Figure 11. Optimization of conditions for the growth of seed AuNP. The role of different components in the growth of seed AuNPs was gauged using UV-Vis spectroscopy by varying their concentrations. UV-Vis spectra of the AuNPs **a** when the concentration of GOx was varied from 35 to 85 U/mL, **b** when the concentration of glucose was varied from 10 to 50 mM, **c** when the concentration of $AuCl_4^-$ was varied from 0.2 to 1 mM, and **d** when the concentration of seed AuNPs was varied from 2 to 8 nM. **e-h** TEM images of grown AuNPs and their conditions are given at the bottom of the images (scale bar is 100 nm).

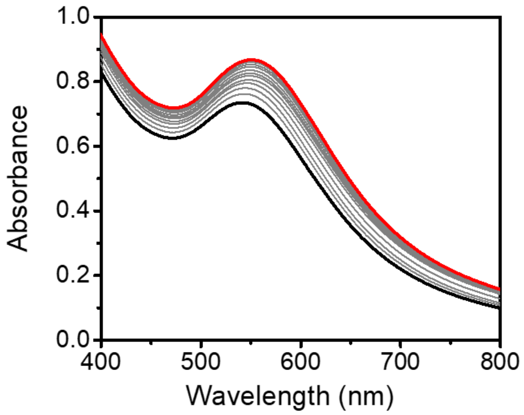

Supplementary Figure 12. Time-dependent UV-Vis spectra of seeded growth of AuNP. Time-dependent UV-Vis spectral changes of a solution containing seed AuNP (8 nM), GOx (35 U/mL), glucose (50 mM), and $AuCl_4^-$ (0.6 mM). UV-Vis spectra were recorded every minute for 11 minutes (starting from the black to the red line).

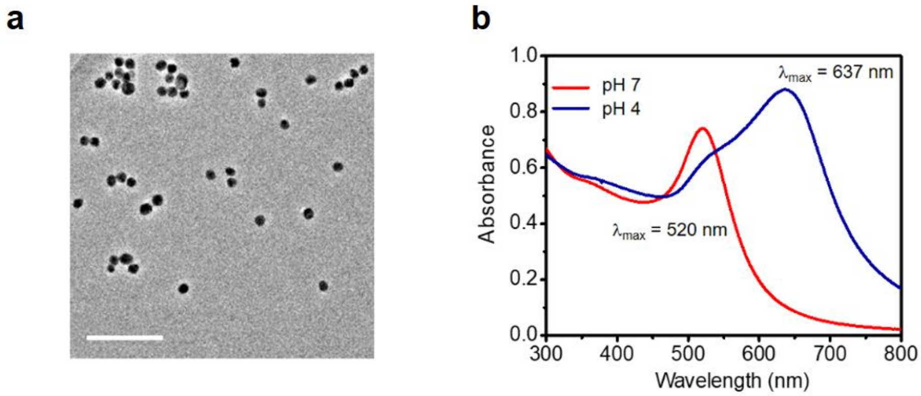

Supplementary Figure 13. Characterization of TA-AuNP. a TEM image of TA-AuNP (scale bar is 100 nm). **b** pH dependent UV-Vis spectral changes of TA-AuNP.

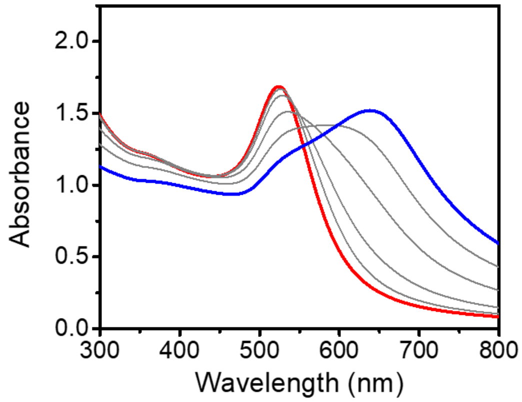

Supplementary Figure 14. Time-dependent UV-Vis spectra of TA-AuNP solution with glucose/GOx reaction system. Time-dependent UV-Vis spectral changes of a solution containing TA-AuNP (6 nM), GOx (40 U/mL), glucose (50 mM), and NaCl (50 mM). UV-Vis spectra were recorded every minute for 5 minutes (starting from the red to the blue line).

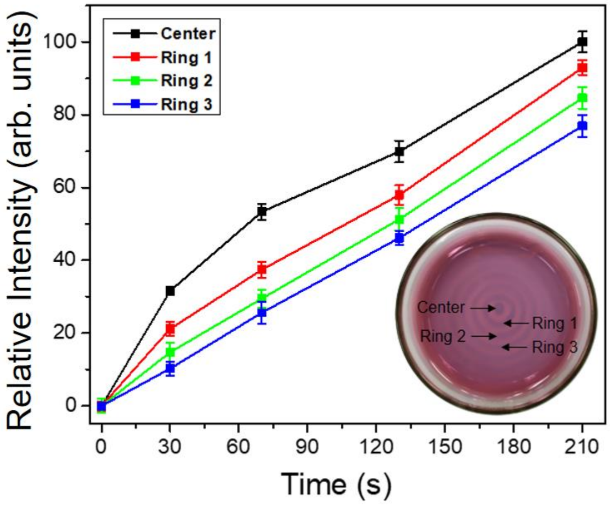

Supplementary Figure 15. Time-dependent analysis for the TA-AuNP concentric ring pattern. Time dependent TA-AuNP aggregation formation was analyzed based on the image intensity with standard deviation (N = 10) from the center to the third ring of the colored pattern.

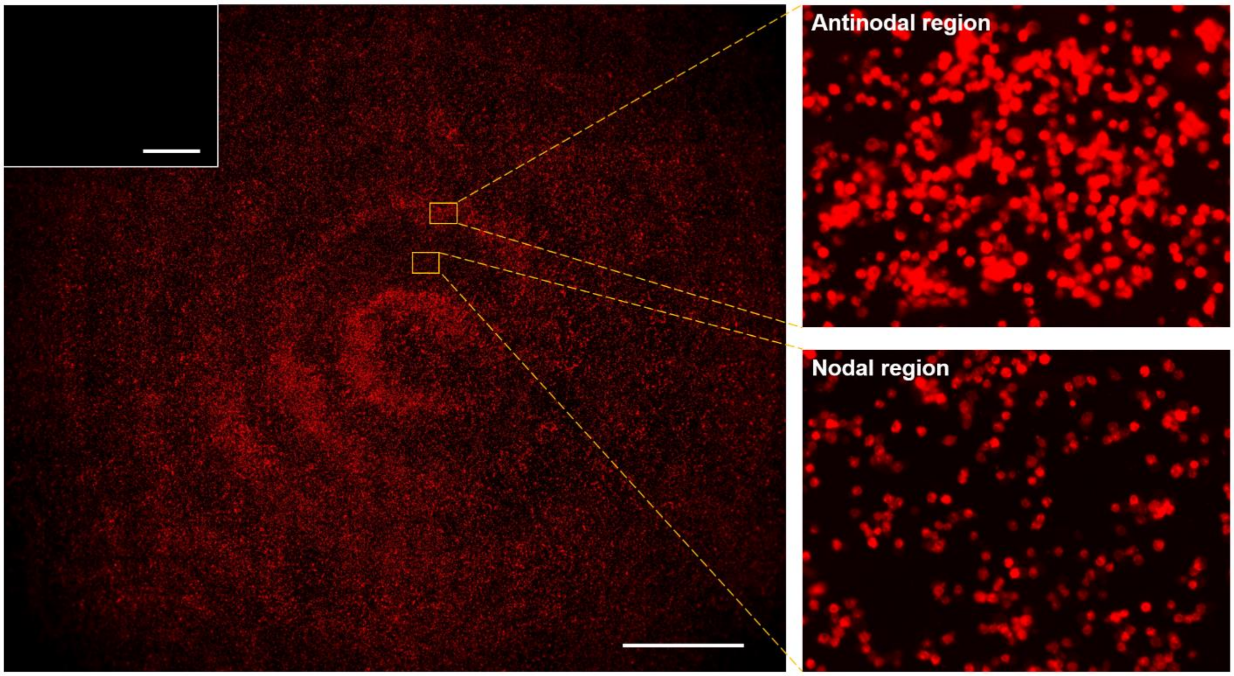

Supplementary Figure 16. Fluorescence microscope images of HeLa cell growth pattern on a patterned hydrogel. The large area image (left, from Fig. 5d, scale bar is 5 mm) shows concentric ring patterns. Comparing the enlarged images (right upper and lower images, respectively), it can be clearly seen that there are much more cells in the antinodal region than in the nodal region. Inset: Fluorescence microscope image of the center part of the patterned hydrogel after treated with a cell-free dye solution. Non-specific adsorption of the dye on the surface was not observed (Scale bar is 2 mm).

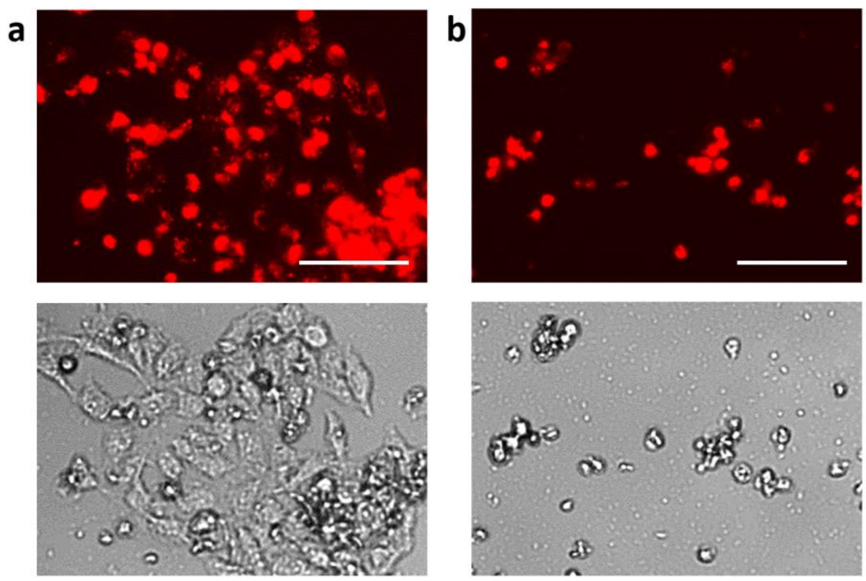

Supplementary Figure 17. HeLa cell images on a patterned hydrogel. a Fluorescence (top) and optical microscope (bottom) images of HeLa cells on the antinodal region. **b** Fluorescence (top) and optical microscope (bottom) images of HeLa cells on the nodal region. It can be clearly seen that well-spread cell morphologies are observed in the antinodal region rather than in the nodal region. Scale bar is 50 μm.

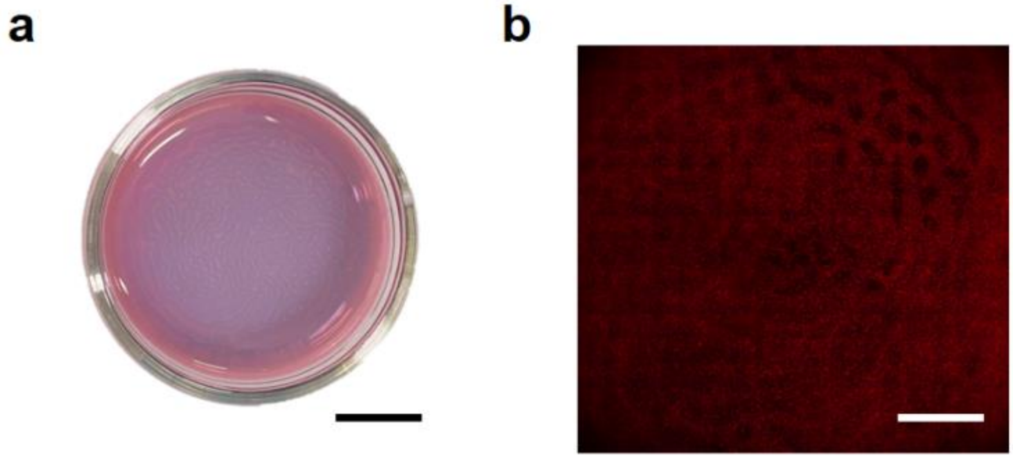

Supplementary Figure 18. Cell growth on a randomly patterned hydrogel. a A photograph of a randomly patterned hydrogel prepared without applying audible sound (scale bar is 10 mm). **b** Fluorescence microscope image of HeLa cell growth pattern on the aforementioned hydrogel (scale bar is 5 mm).

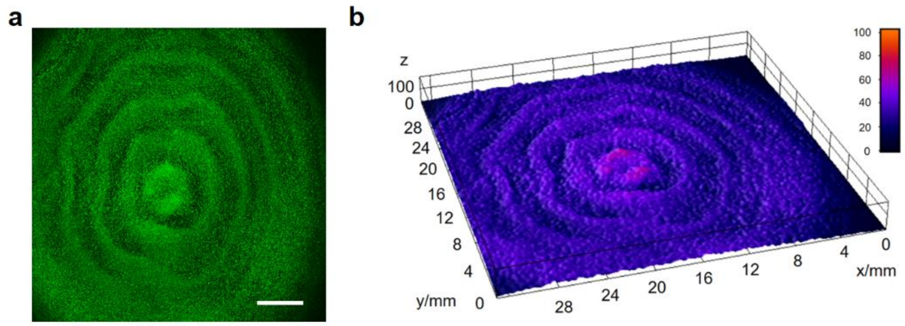

Supplementary Figure 19. Human umbilical vein endothelial cell (HUVEC) growth pattern on a patterned hydrogel. a Fluorescence microscope image of HUVEC growth pattern on a patterned hydrogel (scale bar is 5 mm). **b** 3D fluorescence intensity plot of the region specific cell growth pattern.
